# Hot-Band Anti-Stokes Fluorescence Properties of Alexa Fluor 568

**DOI:** 10.1007/s10895-020-02496-0

**Published:** 2020-02-29

**Authors:** Tamás Gajdos, Béla Hopp, Miklós Erdélyi

**Affiliations:** Department of Optics and Quantum Electronics, University of Szeged, Dóm tér 9., Szeged, 6720 Hungary

**Keywords:** Upconversion, Hot band absorption, Anti-Stokes fluorescence

## Abstract

Hot-band absorption and anti-Stokes emission properties of an organic fluorescent dye, Alexa Fluor 568, were characterized and compared with those of Rhodamine 101. The comparison of the properties (e.g., quantum efficiency, spectral distribution, thermal properties, and fluorescence lifetime) between the two dyes confirms that both dyes undergo the same process when excited in the red spectral region. Possible undesirable crosstalk effects and applications in dSTORM microscopy were demonstrated and discussed.

## Introduction

The emission spectra of fluorescent dyes can be elucidated via radiative/non-radiative transitions between the electronic states and internal conversions. Typically, the process begins with a single photon absorption when the molecule is excited to its higher *S*_1_ or *S*_2_ electronic states from the *S*_0_ ground state. Due to vibrations, the electronic states are broadened; after the photon absorption step, the molecule rapidly relaxes to its lowest vibrational level of *S*_1_ (Kasha’s rule) [1]. Internal conversion is significantly faster (< 1 ps) than the fluorescence lifetime (1-10 ns) of an excited state. During the fluorescence step, the molecule returns to its ground *S*_0_ state and emits a single fluorescence photon. Because of the energy loss due to internal conversion, the emitted photon has a longer wavelength, which is called the Stokes shift [2], and the energy difference is typically converted into heat.

However, during the anti-Stokes fluorescence, the emitted photon has higher energy (lower wavelength) than the excitation photon. This process requires some additional energy. The thermodynamic background of this process was highly debated in 1946 by Pringsheim and Vavilov and was eventually solved by Landau in the same year [3]. Several types of anti-Stokes fluorescence pathways can be separated based on the excitation model and the source of the missing energy. The latter can come from the absorption of multiple photons or from the heat-populated vibrational states. During two-photon absorption process, a virtual state is assumed to exist between *S*_0_ and *S*_1_ to excite the molecule. This is a nonlinear third-order process that was originally predicted in 1931 [4] and experimentally was first demonstrated by laser excitation in 1961 [5]. Because the probability of the two-photon absorption process is low, high energy excitation must be used to ensure the sufficient number of emitted photons.

The triplet-triplet annihilation is a similar process, when the triplet *T*_1_ level is used as an intermediate energy storage state. During this process, two excited molecules undergo energy transfer. One returns to its ground *S*_0_ state while the other one excites to the *S*_1_ state. As a result, the emission spectrum exhibits fluorescence characteristics. This process is also called P-type delayed fluorescence [6, 7].

There are two single photon excitation models that produce anti-Stokes fluorescence. In the first model, molecules that are excited to the *T*_1_ state are considered. Because of the long lifetime of the triplet state, there is a possibility for the molecule to cross into the *S*_1_ state via temperature activation (reverse intersystem crossing). During the relaxation, emission can occur from the *S*_1_ or *T*_1_ states depending on whether the reverse intersystem crossing occurred. Because it was first observed in eosin [8] and the lifetime of the process concurs with phosphorescence, it is called Type-E delayed fluorescence.

In the second single-photon model, the molecule is excited from a higher vibrational state of *S*_0_ to *S*_1_. Because at thermal equilibrium the occupation of the vibrational levels follows the Boltzmann distribution, the possibility of absorption from higher energy levels is low and temperature-dependent. However, the lifetime and emission spectra are not affected. The hot-band absorption has been observed with several organic dyes: Rhodamine 101 (Rh101) [9], Rh6G [10], Rh640 [11], RhB [12], Oxazine 1 [13], and cyanide-type [14]. Because of the nature of the initial state, the first applications were based around laser cooling [9, 15–18]. Here, we report the anti-Stokes fluorescence properties of Alexa Fluor 568 (AF568) when it is excited in the red-edge region of its absorption spectrum. Although AF568 is a widely used fluorescence dye in confocal and super-resolution microscopy, to our knowledge, its anti-Stokes emission properties have not been studied.

**Fig. 1:**
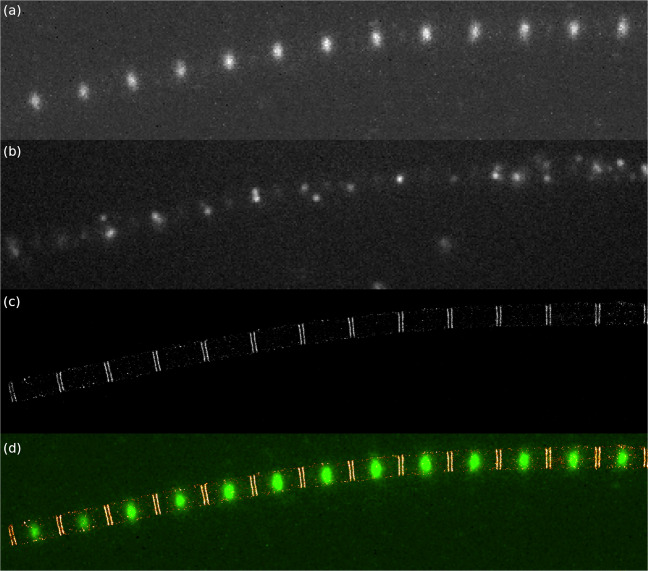
dSTORM measurement of a double-stained drosophila myofibril excited with a 640 nm laser. A total of 10000 frames were acquired in the Dual Gain mode with an exposure time of 20 ms using a single Andor Zyla 4.2 sCMOS detector with Cairn OptoSplit II+Bypass. The sample is labelled with b2-AF568 and tropomyosin-AF647 [20]. **a** In the 600/37 emission channel, the anti-Stokes emission of AF568 is clearly visible in the I-bands (≈ 500 counts). **b** In the captured frame from the 647 high-pass emission channel, the blinking events of the AF647 in H-zone (≈ 1800 counts) and the crosstalk from the AF568 can be seen in the I-band. **c** The double lines of tropomyosin in the H-zone can be seen in the reconstructed super-resolved image. **d** The merged image from (**a**) and (**c**)

Assuming a two-channel two-colour super-resolution dSTORM imaging assay with a sample simultaneously labelled with AF568 and AF647 dyes (Fig. 1), the intensity of the captured anti-Stokes images (in the 600/37 channel) when excited with 640 nm laser is comparable to a weak single molecule fluorescence signal of the AF647 dye in the normal long pass emission range. For overlapping structures, the static anti-Stokes signal can introduce a structured background. Due to the spatial shift of localizations towards the peak of the structured background, the final image can be distorted [19]. To minimize this effect the properties of the AF568 anti-Stokes fluorescence had to be specified. To distinguish between the possible excitation models, the relationships between excitation power, excitation wavelength, and emission intensity were measured.

## Results

### Fluorescence Intensity

First, we determined the function between the excitation power and emitted intensity at different excitation wavelengths (634, 640, and 647 nm). The output power of each source was calibrated and measured after the microscope objective (Nikon CFI Plan Apo Lambda 60x oil). Both Rh101 and AF568 dyes were prepared in a 10^− 4^ M solution of methanol and PBS (1:1). Because of the low emission intensity, an additional bandpass emission filter (Semrock 582/75) was used after the main dichroic mirror (Semrock Di01-R405/488/532/635) and quad-band bandpass emission filter (Semrock FF01-446/510/581/703). For each laser setting, a single frame was captured with an exposure time of 100 ms using an EMCCD camera (Andor iXon 897). The lasers were fibre coupled, collimated, and expanded before they were focused into the back focal plane of the microscope objective. Assuming a Gaussian beam shape in the image plane, two Gaussian distribution curves were fitted on the captured images along each axis. Using the fitted parameters, the volume under the two-dimensional Gaussian distribution was calculated, and the background noise was subtracted. This value was used to indicate the intensity of the anti-Stokes fluorescence.

**Fig. 2:**
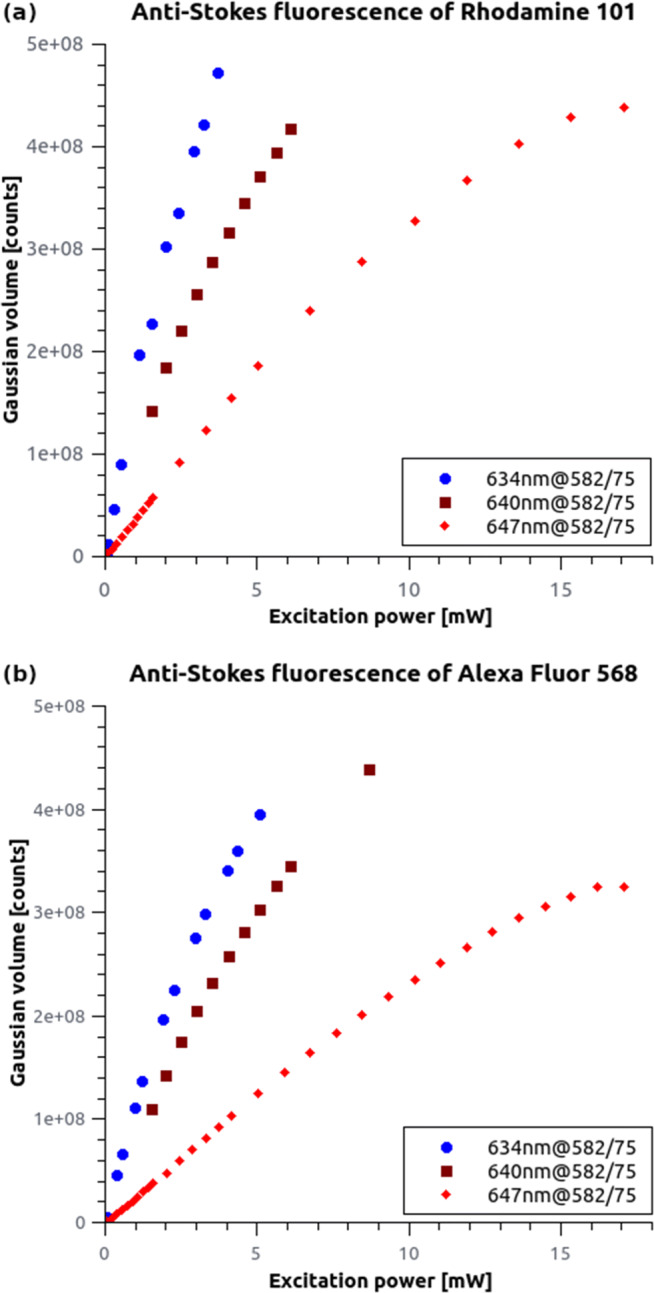
Experimental measurements of the anti-Stokes fluorescence of Rh101 and AF568. The measured signal is clearly dependent on the excitation wavelength and exhibits a near-linear dependence with the excitation power. The divergence can be attributed to the saturation of the camera

Expectedly, the absorption cross section is lower for both dyes when excited with longer wavelengths because there are fewer number of fluorophores with high-enough excitable vibrational states in the excited volume (Fig. 2). The measured intensity shows a linear dependence at the lower excitation power range (< 5 mW). The nonlinear feature at higher excitation power is related to the depletion of the electron multiplying layer of the detector [21]. This effect can be reduced using shorter exposure times. In addition, we observed that the signal can be recorded even below a 1 mW excitation power, and we could not observe any threshold while decreasing the laser power. Both dyes show the same behaviour: the longer the excitation wavelength, the lower the emission rate. Based on these results, it is possible to reject the two-photon absorption model when the emission has a quadratic dependence on the excitation intensity.

### Fluorescence Emission Spectra

In the second experiment, the emission spectra of Stokes and anti-Stokes fluorescence were recorded. Continuous wave laser sources (405 and 634 nm) were used for excitation with two dichroic mirror and emission filter sets (Chroma ZT458rdc + Chroma ET460lp and Semrock Di01-R405/488/532/635 + Semrock FF01-446/-510/581/703). Rh101 and AF568 solutions were prepared in 10^− 2^ M and 10^− 2^ M methanol solutions, respectively. The signal from the sample was coupled into a multimode fibre using a reflective collimator at the camera port and measured with an OceanOptics QE65000 spectrometer.

**Fig. 3:**
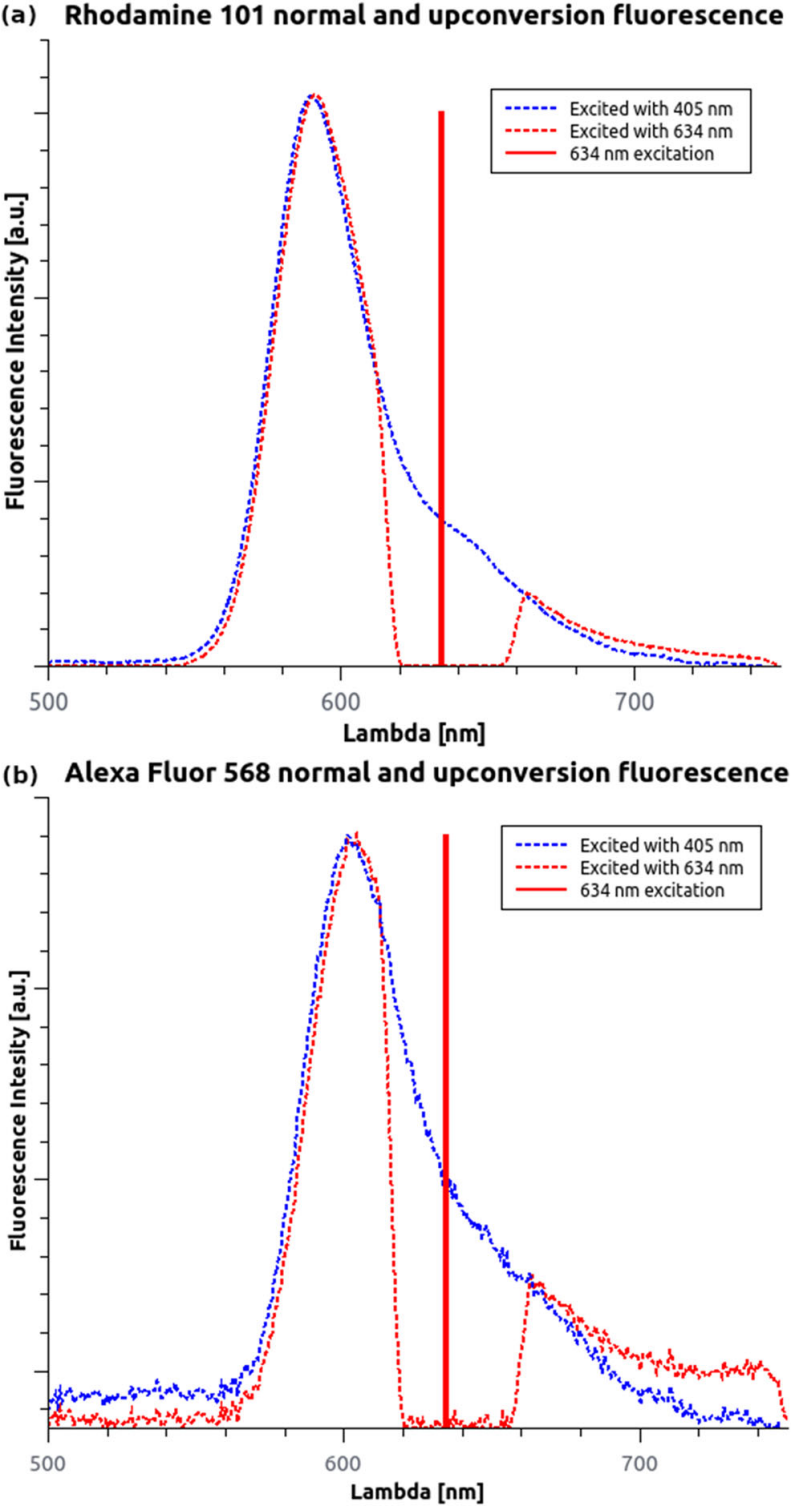
Recorded Stokes- and anti-Stokes fluorescence spectra for Rh101 (**a**) and AF568 (**b**). The additional signal above 700 nm is related to the NIR background from the solution

The results show that the normalized shape of the Stokes and anti-Stokes emission spectra highly overlap (Fig. 3). Only a slight 1–1.5 nm red shift is noticeable under the excitation with longer wavelengths caused by solute-solvent interaction [22–24]. For Rh101 and AF568, the maxima of the anti-Stokes emission spectra were 591 nm and 604 nm, respectively. In addition, a low background was observed in the emission spectra above 700 nm when it was excited at 634 nm. This was reproducible with other wavelengths and even without the fluorescent dye; thus, it was associated with the buffer. No other emission peaks were observed in the measurement range of the spectrometer. It can be assumed that the dominant relaxation path is from *S*_1_ to *S*_0_.

### Temperature-Dependent Fluorescence

A third experiment aimed to measure the temperature-dependence of the absorption cross section. A thermal paste was applied to a glass slide, and the slide was glued to a Peltier-cooled mount (Thorlabs TCLDM9). Each dye sample was prepared in a 10^− 5^ M solution of methanol and PBS (1:1) to minimize the solvent’s evaporation between the slide and cover glass. To thermally isolate the sample from the microscope frame, a dry objective (Nikon CFI S Plan Fluor ELWD 20XC) was used throughout these experiments. For excitation, a 640 nm wavelength pulsed laser with a repetition rate of 20 MHz was used in a confocal line-scanning mode. The focal plane was set to approximately 1 *μ* m above the cover glass and was maintained by the Nikon perfect focus system. The temperature was changed gradually between 12 ^∘^C and 48 ^∘^C with a step size of 4 ^∘^C. After the sample reached thermal equilibrium (typically 120 s), a 60 s measurement was recorded in the two emission channels (600/50 and 690/70). The measurements were repeated several times and averaged. These multiple passthroughs over the entire temperature range in both directions eliminated the possible hysteresis effects and photobleaching. The measurements with AF568 dye were slightly affected by photobleaching after multiple heating–cooling cycles. This effect was not observable for the Rh101 dye.

**Fig. 4:**
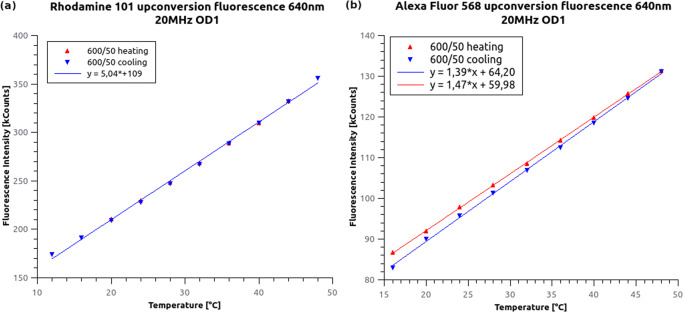
Temperature-dependence of the anti-Stokes fluorescent intensity measured for R101 (**a**) and AF568 (**b**) solutions. Although a linear curve was fitted for the small measured temperature range, the hot-band excitation model predicts a Boltzmann distribution curve

A strong increase in anti-Stokes emission as a function of temperature was observed (Fig. 4). According to the theory [25], it can be assumed that the measured curves follow a Boltzmann statistic [$I(T) = A \cdot e^{(-b/(k_{B} \cdot T))}$], which is similar to the occupancy of the vibrational states in a single electronic band. However, we observed a linear dependence because the measurements were performed at room temperature and in a limited thermal range. The steepness of the fitted line onto the measured data was concentration-dependent because it was connected to the number of molecules in the excitation volume. The experiment was also repeated with a 560 nm wavelength excitation in the same temperature range used for AF568. However, the recorded intensity did not show any temperature-dependence. This observation is in good agreement with the hot-band excitation model. However, to discard the delayed fluorescence models, an additional experiment was carried out.

### Fluorescence Lifetime Measurements

Fluorescence lifetimes for Stokes and anti-Stokes emissions were measured with 560 nm and 640 nm wavelength excitations. To avoid nonlinear effects and detector saturation, neutral filters with OD3 and OD1 were used in the excitation path. The repetition rate of the pulsed laser sources was set to 20 MHz. The samples from the first experiment were reused, and four FLIM measurements were recorded in the 600/50 emission channel. The acquisition was stopped when the collected number of emitted photons exceeded 10^5^. The arrival time distribution was fitted with a single exponential model using the Tail Fit method (SymPhoTime 64, PicoQuant).

**Fig. 5:**
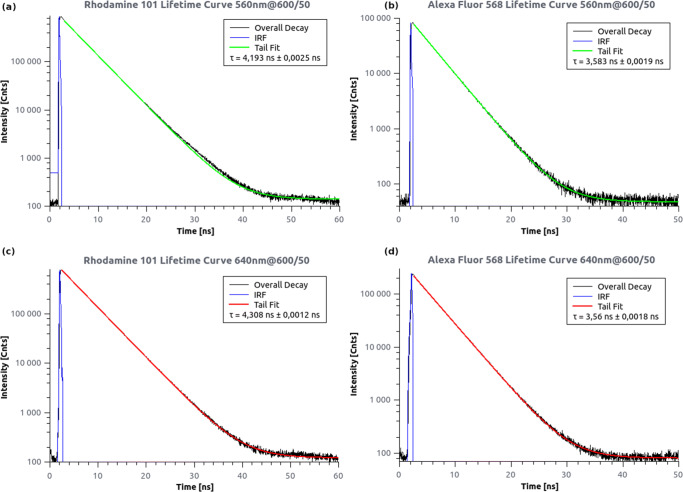
Stokes and anti-Stokes fluorescent lifetime curves for Rh101 (**a**, **c**) and AF568 (**b**, **d**). A single exponential tail-fit model was used to determine the fluorescent lifetimes for each recorded arrival time

The fitted lifetimes for the anti-Stokes fluorescence (4.193 ns; 3.56 ns) were found to be consistent with those for the Stokes fluorescence (4.308 ns; 3.56 ns) (Fig. 5). This observation discards any delayed fluorescence models. When measuring the anti-Stokes lifetime, a slight broadening of the fitted instrument response function was observed. This may indicate a very fast (< 1 ns) relaxation path, where no intersystem crossing occurs.

## Potential Applications

The spectral, lifetime and temperature dependent measurements have proved that the emitted anti-Stokes fluorescence is identical to regular Stokes shift fluorescence, therefore separation of these signals pose a challenge. In multicolour fluorescence microscopy, especially when high excitation power is applied, the reduction of spectral crosstalk is essential. AF568 is a frequently applied second dye in the dSTORM technique [26] besides AF647. Capturing image stacks at high (> 3 kW/c*m*^2^) laser power in the far-red region can significantly increase the fluorescence background in the ”green channel”. This undesirable effect is typically mitigated with additional emission filters. However, the problem can be dealt with on the excitation side too, either by choosing an excitation laser with a higher wavelength (Fig. 2) or by reducing the sample’s temperature (Fig. 4). In the first case the number of excitable vibrational states, while in the second one the occupancy of the vibrational states is lowered. However, anti-Stokes fluorescence can be used in multiple ways when captured in a separate spectral window (600/50) simultaneously with the main emission channel (690/70) (e.g. Cairn OptoSplit II, or Hamamatsu W-View Gemini). Here we discuss three potential application areas of the anti-Stokes fluorescence of AF568.

During dSTORM super-resolution measurements the lateral and axial drift can reduce the final image quality. The axial drift is typically minimized by using focus keeping hardware, and the lateral one can only be removed with software-based solutions (using fiducial markers or by correlating the localizations in time). However, the density and spatial distribution of fiducial markers on the sample cannot be controlled. They also introduce a high intensity light source, precluding the localization of adjacent single dye emitters. Fluorescent beads labelled with hot-band active dyes (e.g. Rh101 or AF568) can be used to address this issue. The lateral drift can be corrected by simultaneously recording the anti-Stokes signal in a separate spectral channel. A similar approach using only one emission window has been already proposed [27]. In addition, the approach can be further developed to correct the axial drift in astigmatic 3D localization microscopy, when the elliptical shape of the PSF provides information on the axial position.

The reduced FOV (< 20 × 20 μ*m*^2^) and finite lifetime of the switching buffer (< 4 hours) in localization based microscopy methods make the selection of ROI a difficult task. Labelling the interesting cells, cell components, structures or proteins in the sample with a different dye can be used to significantly reduce searching time. Hot-band active dyes are an ideal choice for such applications since the two spectral channels can be captured simultaneously using the same laser source. For example, the axon initial segment of a neuron in a brain tissue experiment can be found by labelling the βIV spectrin [28]. In a muscle cell culture desmin accumulation is a key indicator for differentiation [29, 30], while in myofibrils the elastic protein components (kettin, Sls700 etc.) related to the I-band can be marked [20].

Autofluorescence is a critical limitation in fluorescence microscopy. It reduces the signal-to-noise ratio and can introduce artefacts. Several sample preparation protocols and histochemical techniques [31] have been proposed to eliminate or reduce such undesirable effects. Since autofluorescence is typically more significant at the low wavelength range (due to the high photon energy), the application of far-red dyes can be a possible solution too. However, hot-band active dyes can also be used for reducing autofluorescence. Samples labelled with AF568 and excited by red lasers (e.g. 647 nm) can be imaged with reduced autofluorescence signal. In this case only the hot-band emission signal of the fluorescent dye can be captured [32], and the autofluorescence background in the far-red region can be spectrally separated (see Fig. 3). A similar signal-to-noise ratio enhancement has been already demonstrated via upconverting nanoparticles [33, 34].

## Conclusion

Comparative time-resolved and steady-state measurements of the anti-Stokes fluorescence emission of AF568 have revealed that the emission intensity linearly depends on both the excitation power and temperature in the studied ranges. No intensity threshold was detected and both the emission spectrum and the fluorescence lifetime were found to be identical to the normal Stokes fluorescence values. These findings have confirmed that the detected anti-Stokes fluorescence of AF568 is the consequence of the hot-band absorption-emission pathway. The undesirable anti-Stokes emission rate can be reduced by manipulating either the temperature or the excitation wavelength. However, several potential applications have been suggested where hot-band active dyes can be used for drift correction, fast ROI selection or autofluorescence reduction.
